# *Ginkgo biloba* leaf extract induces DNA damage by inhibiting topoisomerase II activity in human hepatic cells

**DOI:** 10.1038/srep14633

**Published:** 2015-09-30

**Authors:** Zhuhong Zhang, Si Chen, Hu Mei, Jiekun Xuan, Xiaoqing Guo, Letha Couch, Vasily N. Dobrovolsky, Lei Guo, Nan Mei

**Affiliations:** 1 Division of Genetic and Molecular Toxicology, National Center for Toxicological Research, Jefferson, AR 72079, USA.; 2 Division of Biochemical Toxicology, National Center for Toxicological Research, Jefferson, AR 72079, USA; 3 College of Bioengineering, Chongqing University, Chongqing 400044, China

## Abstract

*Ginkgo biloba* leaf extract has been shown to increase the incidence in liver tumors in mice in a 2-year bioassay conducted by the National Toxicology Program. In this study, the DNA damaging effects of *Ginkgo biloba* leaf extract and many of its constituents were evaluated in human hepatic HepG2 cells and the underlying mechanism was determined. A molecular docking study revealed that quercetin, a flavonoid constituent of *Ginkgo biloba*, showed a higher potential to interact with topoisomerase II (Topo II) than did the other *Ginkgo biloba* constituents; this *in silico* prediction was confirmed by using a biochemical assay to study Topo II enzyme inhibition. Moreover, as measured by the Comet assay and the induction of γ-H2A.X, quercetin, followed by keampferol and isorhamnetin, appeared to be the most potent DNA damage inducer in HepG2 cells. In Topo II knockdown cells, DNA damage triggered by *Ginkgo biloba* leaf extract or quercetin was dramatically decreased, indicating that DNA damage is directly associated with Topo II. DNA damage was also observed when cells were treated with commercially available *Ginkgo biloba* extract product. Our findings suggest that *Ginkgo biloba* leaf extract- and quercetin*-*induced *in vitro* genotoxicity may be the result of Topo II inhibition.

*Ginkgo biloba* leaves and their extracts have been used to treat a variety of ailments, including memory loss and cognitive disorders, arrhythmias and ischemic heart disease, diabetes, skin infection, cancer, and thromboses^1^,^2^. *Ginkgo biloba* leaf extracts are widely available in herbal supplement stores and online in the United States^3^, and it is one of top-selling botanicals with total sales of $94 million annually^4^. Since *Ginkgo biloba* leaf extract and some of its active ingredients demonstrate biological activities, it may be consumed in large doses for an extended period of time. In addition, some of *Ginkgo biloba*’s ingredients possess mutagenic activities. For such reasons, *Ginkgo biloba* leaf extract was nominated to the National Toxicology Program (NTP) for toxicological evaluation and a 2-year carcinogenicity bioassay. Administration of *Ginkgo biloba* leaf extract demonstrated carcinogenic activity in livers of mice^5^. Because the mechanism of *Ginkgo biloba*-caused liver carcinogenicity is not well understood, it was of interest to study the potential mechanisms and also to identify the constituents that may contribute to its carcinogenicity. In our previous study using the mouse lymphoma cells, we demonstrated that *Ginkgo biloba* leaf extract and some of its constituents (including quercetin and keampferol) induced genotoxicity and DNA damage^6^.

Flavonoids (such as quercetin, kaempferol, and isorhamnetin) and terpenoids (including ginkgolide A, ginkgolide B, ginkgolide C, and bilobalide) are the two major classes of components found in *Ginkgo biloba*^2^,^7^. It has been reported that some flavonoids, with variable chemical structures, target DNA topoisomerases and interrupt the process of DNA replication, and thus hold therapeutic promise for the treatment of cancers^8^. DNA topoisomerases are the enzymes that control DNA topology by breaking and rejoining the sugar-phosphate backbone of DNA^9^,^10^. Topoisomerase I (Topo I) introduces a transient breakage in a single strand of DNA, while Topo II introduces transient breakage in both strands of DNA; both types of breaks are important for DNA replication and cell proliferation during the normal cell cycle. Inhibition of topoisomerase enzyme activity can generate both single and double strand DNA breaks by blocking the ligation step, resulting in significant genotoxicity and cytotoxicity^11^. Prolonged inhibition of topoisomerases leads to unrepaired DNA breaks, thus resulting in genotoxic effects such as multilocus deletions, chromosomal translocations, and loss of heterozygosity. DNA topoisomerase inhibitors are agents designed to inhibit the formation of Topo-DNA complexes by interacting with the enzyme and blocking subsequent steps in the catalytic cycle of the enzymes^12^,^13^,^14^. Topoisomerase inhibitors can be largely classified into two groups based on the specific enzyme inhibited, i.e., Topo I inhibitor and Topo II inhibitor.

Topoisomerase inhibitors display some therapeutic potential, such as anticancer drugs and anti-infective drugs; however, side effects have also been reported^15^. An association between the use of topoisomerase inhibitors and secondary tumor formation has been described, and increased maternal consumption of flavonoids (some are topoisomerase inhibitors) during pregnancy may be associated with infant acute leukemia^16^,^17^,^18^,^19^. A number of environmental chemicals (including pesticides and some agricultural or industrial chemicals) also act as topoisomerase inhibitors in the environment, and these chemicals form a unique group of environmental mutagens and carcinogens with different modes of action associated with their topoisomerase suppression effect^20^,^21^.

In the present study, using a combination of *in silico* tools and biochemical and molecular approaches, we studied the DNA damaging effect caused by *Ginkgo biloba* leaf extract and seven of its constituents in human hepatic HepG2 cells, and examined the seven *Ginkgo biloba* constituents for their potential to interact with topoisomerases. We also investigated the involvement of topoisomerases in *Ginkgo biloba* leaf extract- or quercetin-triggered DNA damage in cells where topoisomerase was silenced.

## Results

### Genotoxicity of *Ginkgo biloba* leaf extract

In our previous study, a 4-h treatment with *Ginkgo biloba* leaf extract induced DNA damage and mutations in the mouse lymphoma cells at the concentration of 1,000 μg/ml and had pro-oxidative effects at concentrations over 400 μg/ml^6^. To confirm the genotoxicity of *Ginkgo biloba* leaf extract in other cell lines, we initially conducted a GreenScreen HC assay by the flow cytometry method that is a human lymphoblastoid TK6 cell-based assay for measuring the cytotoxicity and genotoxicity^22^. In the absence of metabolic activation, a 46-h treatment with *Ginkgo biloba* leaf extract at the concentration of 625 μg/ml reduced the relative cell survival to less than 90% of the vehicle control, indicating a positive response for cytotoxicity (Supplementary Fig. 1A). *Ginkgo biloba* leaf extract at the concentrations of 156–625 μg/ml induced GFP fluorescence above the significance threshold (i.e., 30% induction over and above the baselines of the control cells). The lowest effective concentration was 156 μg/ml (Supplementary Fig. 1B).

**Figure 1:**
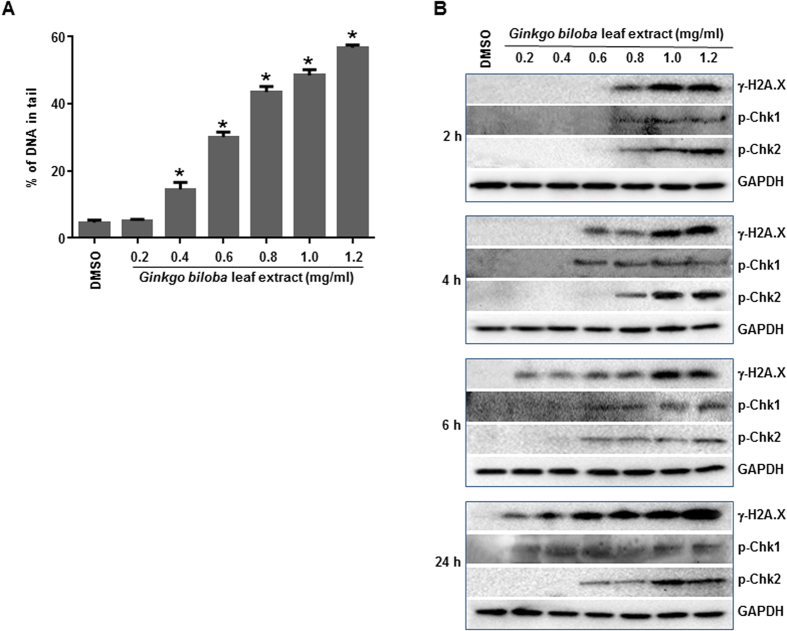
DNA damage induced by *Ginkgo biloba* leaf extract in HepG2 cells. (**A**) HepG2 cells were exposed to increased concentrations (0.2–1.2 mg/ml) of *Ginkgo biloba* leaf extract for 4 h. The data points represent the means ± S.D. for three independent experiments and asterisk indicates *p* < 0.05 when the treatment group was compared with the concurrent control. (**B**) HepG2 cells were exposed to *Ginkgo biloba* leaf extract for 2, 4, 6, and 24 h. Total cellular protein was extracted and levels of γ-H2A.X, p-Chk1, and p-Chk2 were detected by Western blot analysis. GAPDH was used as a loading control. Similar results were obtained from three independent experiments.

We then determined if *Ginkgo biloba* leaf extract induces DNA damage in human hepatic cells, because *Ginkgo biloba* leaf extract has been shown to increase an incidence in liver tumors in mice in an NTP 2-year bioassay^5^. DNA damage was measured by the Comet assay and by the induction of phosphorylation of histone H2A.X (γ-H2A.X) using HepG2 cells. In the standard Comet assay, *Ginkgo biloba* leaf extract resulted in the induction of DNA damage in a concentration-dependent manner, with a significant difference being observed at concentrations above 400 μg/ml (Fig. 1A). The induction of γ-H2A.X, which occurs after DNA double strand breaks and is generally considered as a hallmark of DNA damage^23^, was investigated by measuring γ-H2A.X at Ser139 by Western blot analysis. In addition, DNA damage-responsive cell cycle checkpoint-related proteins, phosphorylated-Chk1 (p-Chk1) and phosphorylated-Chk2 (p-Chk2), were also examined using Western blots. *Ginkgo biloba* leaf extract induced DNA damage in HepG2 cells as demonstrated by a concentration- and time-dependent increase in γ-H2A.X, accompanied by cell cycle perturbation that was evidenced by enhanced expression of both p-Chk1 and p-Chk2 (Fig. 1B).

**Figure 2:**
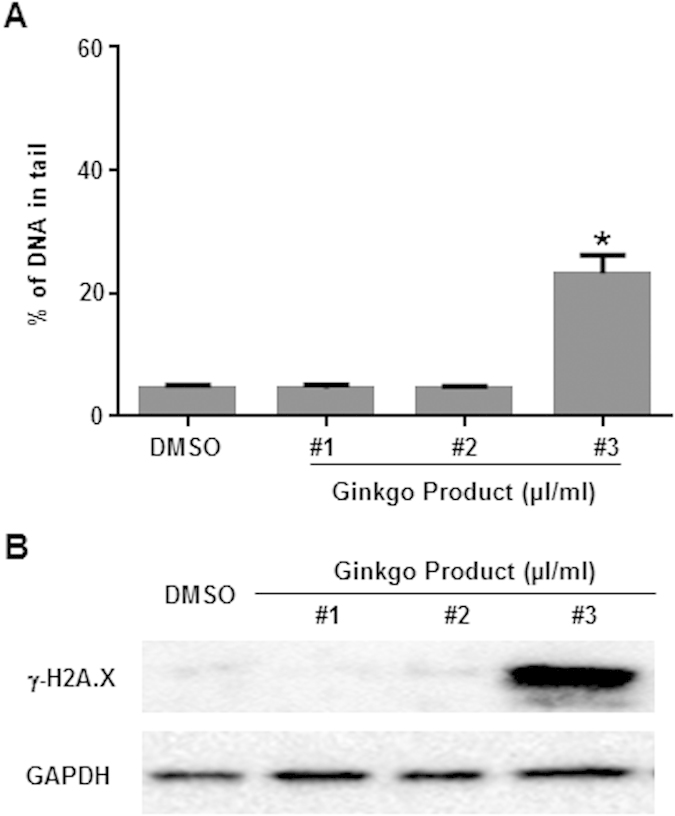
DNA damage induced by some commercial *Ginkgo biloba* extract products in HepG2 cells. HepG2 cells were exposed to 10 μl of each commercial extract product in 10 ml medium for 4 h. (**A**) Data points represent the means ± S.D. for three independent experiments, and asterisk indicates *p* < 0.05 when compared with the concurrent control. (**B**) Total cellular protein was extracted and level of γ-H2A.X was detected by Western blotting with GAPDH as a loading control. Similar results were obtained from three independent experiments.

Since a variety of *Ginkgo biloba* extract products are commercially available in the United States, we randomly selected a *Ginkgo biloba* extract products from three different U.S. companies for testing. We determined whether or not these commercial products produce DNA damage in hepatic cells using the Comet assay and γ-H2A.X as the indicator of DNA strand breaks. Ten μl of each *Ginkgo biloba* extract product was added to separate cultures of HepG2 cells growing in 10 ml medium (final concentration 0.1%, v/v) for 4 h. An increased percentage of DNA in tail and induction of γ-H2A.X was observed in Product #3 (Fig. 2), whereas Products #1 and #2 did not induce a detectable increase in the Comet assay and γ-H2A.X.

### The potential interacting of *Ginkgo biloba* constituents with Topo II

**Figure 3:**
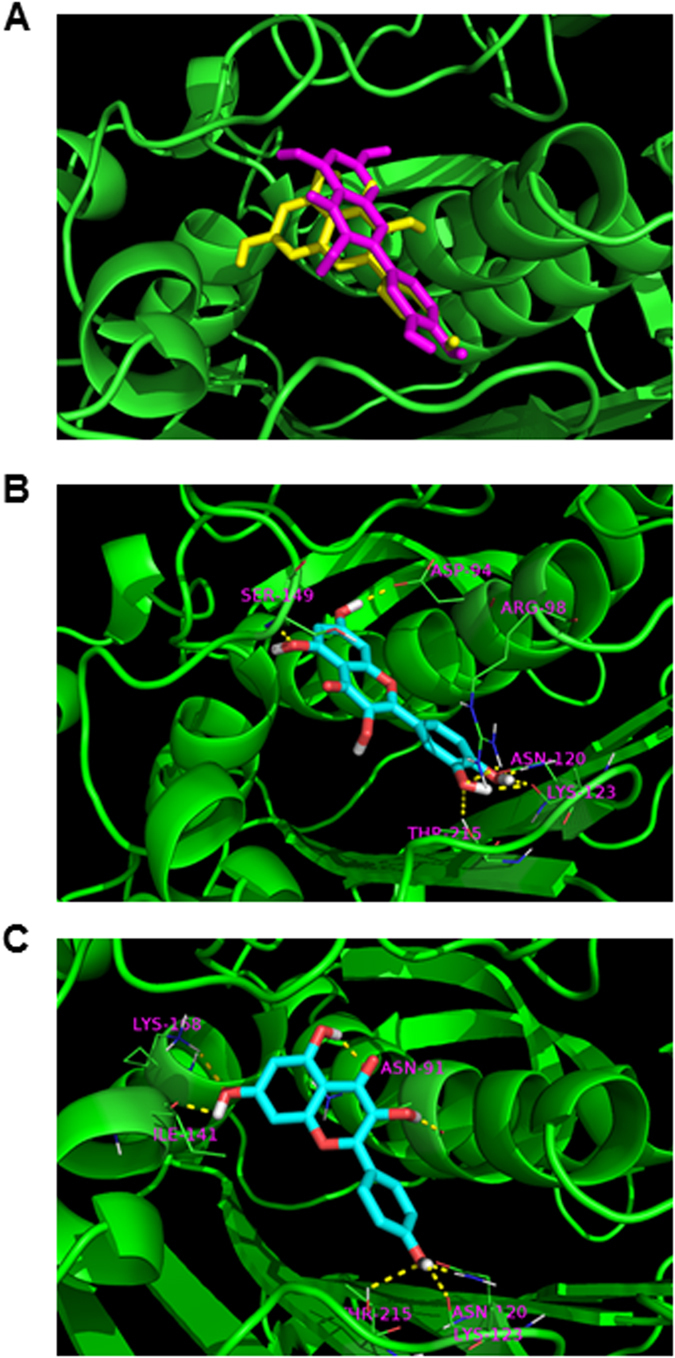
Interaction of Topo II with quercetin and kaempferol. (**A**) The superimposition of quercetin (purple) and kaempferol (yellow) in the binding site of Topo II. The Topo II protein backbone is displayed. (**B**) The H-bond interactions between quercetin and the binding site of Topo II. (**C**) The H-bond interactions between kaemferol and the binding site of Topo II.

Previously, we evaluated the genotoxicity of *Ginkgo biloba* leaf extract and eight of its constituents in mouse L5178Y cells and found that *Ginkgo biloba* leaf extract and two flavonoid constituents, quercetin and kaempferol, are mutagenic due to the induction of DNA double-strand breaks^6^. Considering that some flavonoids target DNA topoisomerases and interrupt the process of DNA replication and that Topo II introduces transient breakage in double strands of DNA, we determined the binding potentials for *Ginkgo biloba*’s constituents to Topo II using molecular docking. Seven constituents of *Ginkgo biloba* (quercetin, kaempferol, isorhamnetin, ginkgolide A, ginkgolide B, ginkgolide C, and bilobalide; chemical structures are shown in Supplementary Fig. 2) were docked into the binding pocket of Topo II (Fig. 3) and the docking scores were calculated (Table 1). Three chemicals had Surflex-dock scores >6.0 with quercetin being the highest (7.39), followed by kaempferol (6.69) and isorhamnetin (6.18). For the other 4 chemicals, the Surflex-dock scores range from 3.74 to 4.82. This result suggests that quercetin, kaempferol, and isorhamnetin have relatively higher potentials to bind to the Topo II. For a consensus evaluation, a *C*-score (0–5) was also calculated and the larger value of *C*-score represents the better consensus. In our calculation, quercetin and isorhamnetin reached 4 (Table 1). The high *C*-score further indicates that quercetin has the highest potential to interact with Topo II. As shown in Fig. 3B, a total of 7H-bonds are formed between quercetin and amino acids Asp94, Ser149, Arg98, Asn120, Lys123 (2 H-bonds), and Thr215. Seven H-bonds are formed between kaempferol and Asn91 (2 H-bonds), Lys168, Ile141, Asn120, Lys123, and Thr215 (Fig. 3C). It is worth noting that Asn120, Lys123, and Thr215 are important for determining the orientations of both quercetin and kaempferol. In addition, hydrophobic interaction is also an important factor affecting the binding activity. The binding pocket of Topo II contains hydrophobic bottom, which are suited for the binding of quercetin and kaempferol with hydrophobic aromatic rings.

### Inhibitory effects of *Ginkgo biloba* constituents on Topo II enzymatic activity

**Figure 4:**
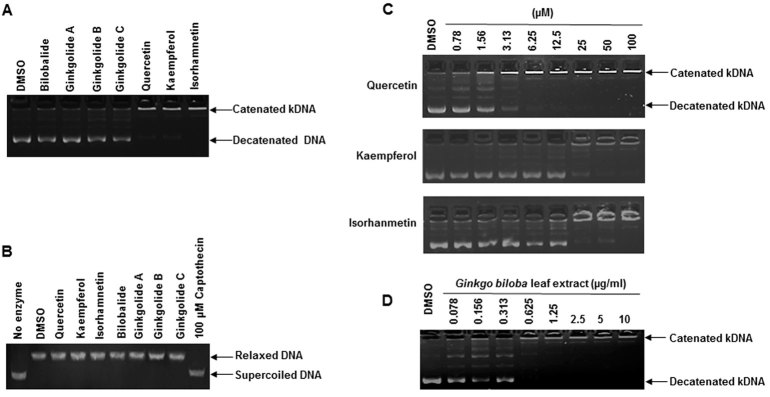
Inhibitory effects of the constituents of *Ginkgo biloba* leaf extract on topoisomerase activities. Using the kDNA decatenation assay (**A,C,D**), kDNA was incubated at 37 °C for 30 min with or without Topo II enzyme in the presence of the indicated *Ginkgo biloba* constituents ((**A**), 500 μM); increasing concentrations of quercetin, kaempferol, and isorhamnetin ((**C**), 0.78–100 μM); or *Ginkgo biloba* leaf extract ((**D**), 0.078–10 μg/ml). Using the supercoiled DNA relaxation assay (**B**), supercoiled pBR322 plasmid DNA was incubated at 37 °C for 30 min with or without Topo I enzyme in the presence of the indicated *Ginkgo biloba* constituents (1 mM); and Camptothecin (100 μM) was used as a Topo I positive control inhibitor.

To validate experimentally the results predicted by the *in silico* studies, the effects of *Ginkgo biloba* constituents on Topo II enzymatic activity were investigated in a cell-free system. Topo II activity was measured by detecting human Topo IIα’s ability to relax double-stranded catenated kinetoplast DNA (kDNA) into decatenated relaxed products. Among the seven constituents tested, quercetin, kaempferol, and isorhamnetin showed inhibitory effects on Topo II (i.e., reducing the amount of decatenated DNA and increasing the amount of catenated DNA), whereas ginkgolide A, ginkgolide B, and ginkgolide C, and bilobalide did not show Topo II inhibitory effects up to a concentration of 500 μM when compared to DMSO (Fig. 4A).

Since previous studies suggested that quercetin and kaempferol may be inhibitors of Topo I^24^,^25^,^26^, it was of interest to investigate whether or not any constituent of *Ginkgo biloba* has Topo I inhibitory potential in a cell-free system. Topo I activity was assayed by measuring the ability of purified human Topo I protein to convert supercoiled plasmid DNA pBR322 to relaxed DNA by cleaving one strand of the duplex DNA. As shown in Fig. 4B, all seven constituents, up to a concentration of 1 mM, showed no inhibitory effects on Topo I, whereas 100 μM captothecin (a well-known Topo I inhibitor) suppressed Topo I relaxation activity (as judged by a decrease in the fraction of relaxed DNA and an increase in supercoiled DNA).

Because none of seven *Ginkgo biloba* constituents inhibited Topo I (Fig. 4B), further analysis was focused on Topo II. As shown in Fig. 4C, three constitutes (quercetin, kaempferol, and isorhamnetin) demonstrated concentration-dependent inhibition of Topo II. Quercetin showed a stronger inhibitory effect, beginning at as low as 3.13 μM, whereas kaempferol and isorhamnetin showed relatively low inhibitory effects (Fig. 4C). Taken together, these results clearly demonstrated that some constituents of *Ginkgo biloba* inhibited Topo II enzyme activity in a concentration-dependent manner, with inhibition potency in the order of quercetin >kaempferol ≈ isorhamnetin ≫ ginkgolide A, ginkgolide B, ginkgolide C, and bilobalide.

*Ginkgo biloba* leaf extract was also examined for its Topo II inhibiting effect in the cell-free system. An inhibitory effect of *Ginkgo biloba* leaf extract on Topo II was observed at a concentration of 0.625 μg/ml and complete inhibition occurred at 2.5 μg/ml (Fig. 4D). It should be noted the concentration of quercetin in 2.5 μg/ml *Ginkgo biloba* leaf extract is approximately 1.62 μM, which is near the minimum concentration at which quercetin inhibits Topo II (Fig. 4C).

### *Ginkgo biloba* constituents induce DNA damage in HepG2 cells

**Figure 5:**
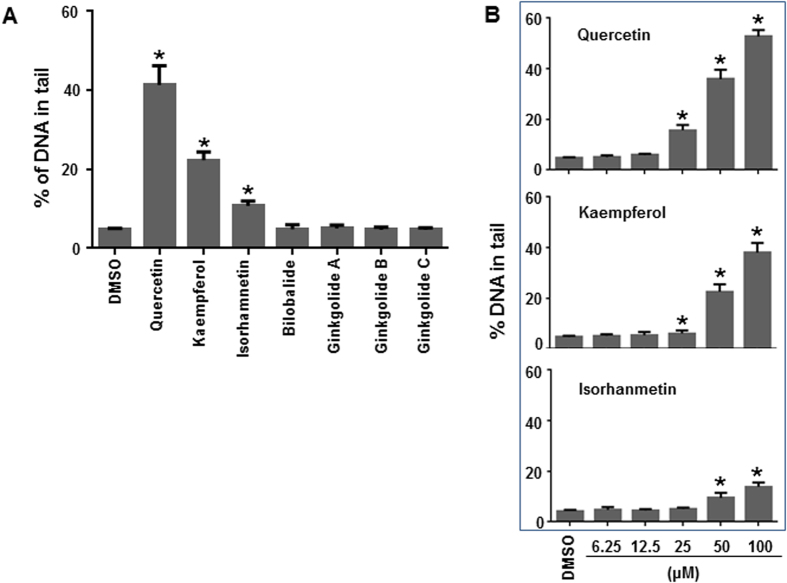
DNA damage induced by three constituents of *Ginkgo biloba* extract in HepG2 cells. HepG2 cells were exposed to seven constituents (50 μM) of *Ginkgo biloba* extract (**A**) or increased concentrations (6.25–100 μM) of quercetin, kaempferol, or isorhamnetin (**B**) for 4 h. The data points represent the means ± S.D. for three independent experiments, and asterisk indicates *p* < 0.05 when the treatment group was compared with the concurrent control.

The DNA damaging potential of seven *Ginkgo biloba* constituents was determined by the Comet assay. HepG2 cells were treated with quercetin, kaempferol, isorhamnetin, ginkgolide A, ginkgolide B, ginkgolide C, or bilobalide at 50 μM for 4 h. Of the constituents tested, quercetin caused the strongest induction of Comet tails (i.e., an increase in DNA breakage) with a tail intensity of 41%, followed by kaempferol (22% tail intensity) and isorhamnetin (11% tail intensity), whereas the other four compounds (ginkgolide A, ginkgolide B, ginkgolide C, and bilobalide) did not show significant DNA damaging effects (Fig. 5A). Concentration-dependent DNA strand breakage by quercetin, kaempferol, or isorhamnetin was further examined with a 4-h treatment; all three constituents caused a concentration-dependent increase in the Comet tail intensity (Fig. 5B). Significant DNA damage was observed at the concentration of ≥25 μM for quercetin and kaempferol, while isorhamnetin caused significant DNA breakage at the concentration of ≥50 μM.

**Figure 6:**
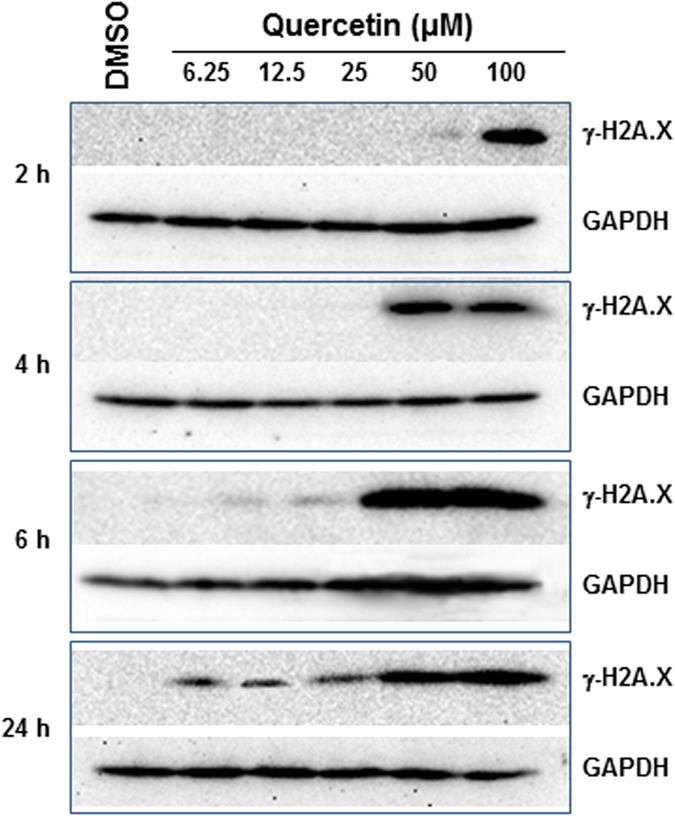
DNA damage induced by quercetin in HepG2 cells. HepG2 cells were exposed to increasing concentrations (6.25–100 μM) of quercetin for 2, 4, 6, and 24 h. Total cellular protein was extracted at the indicated times and concentrations, and levels of phosphorylated (γ-H2A.X) were detected by Western blotting. GAPDH was used as a loading control. Similar results were obtained from three independent experiments.

Quercetin has the highest potency for both Topo II inhibition and DNA damaging activity, compared to other *Ginkgo biloba* constituents tested, and quercetin is likely the major contributor to the genotoxicity caused by *Ginkgo biloba* leaf extract as demonstrated in our previous study^6^; we thus focused on quercetin for a detailed mechanistic study (Fig. 6). γ-H2A.X was induced in the cells treated with 100 μM quercetin as early as 2 h, and the induction was observed at lower concentrations at longer time treatments; for instance, γ-H2A.X was detected in the cells treated with 6.25 μM quercetin after a 24-h treatment. These results indicate the induction of phosphorylated H2A.X is time- and concentration-dependent.

### Quercetin disturbs cell cycle and activates checkpoint-related proteins in HepG2 cells

**Figure 7:**
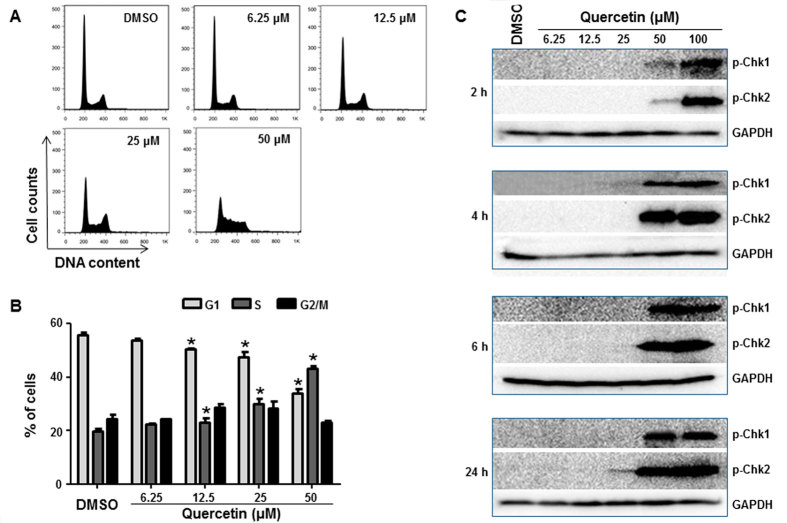
Effects of quercetin on the cell cycle of HepG2 cells. (**A**) Histograms show DNA content analyses for HepG2 cells treated with the indicated concentrations of quercetin for 24 h by flow cytometric analysis. Treated cells were stained with propidium iodide (PI) and processed for cell cycle analysis. (**B**) The bar graph depicts the mean percentage of each cell cycle phase ± S.D. from four independent experiments. *p < 0.05 representing significant difference from the vehicle control. (**C**) Expression of cell cycle checkpoint-related proteins was determined in HepG2 cells treated with the indicated concentrations of quercetin for 2, 4, 6, and 24 h. Treated cells were lysed and subjected to Western blot analyses with antibodies against phospho-Chk1 and phospho-Chk2.

HepG2 cells were treated with various concentrations of quercetin for 24 h (no significant cytotoxicity was observed under these conditions, Supplementary Fig. 3) and cell cycle profiles were analyzed by measuring the DNA content using flow cytometry (Fig. 7A). Cell cycle distribution analysis indicated that there was a concentration-dependent increase in the number of cells in S-phase (Fig. 7B). Concomitantly, there was a decrease in the number of cells in G1-phase and no significant change in G2/M-phase. These results suggest that quercetin disturbs cell cycle progression, leading to the accumulation of cells in S-phase. In addition, we characterized the DNA damaging effect and cell cycle arrest induced by quercetin. Treatment of HepG2 cells with quercetin resulted in an increase in p-Chk1 and p-Chk2, and the induction of both checkpoint-related proteins was observed as early as 2 h (Fig. 7C).

### Knockdown of the Topo II gene decreases DNA damaging effect caused by *Ginkgo biloba* leaf extract or quercetin

**Figure 8:**
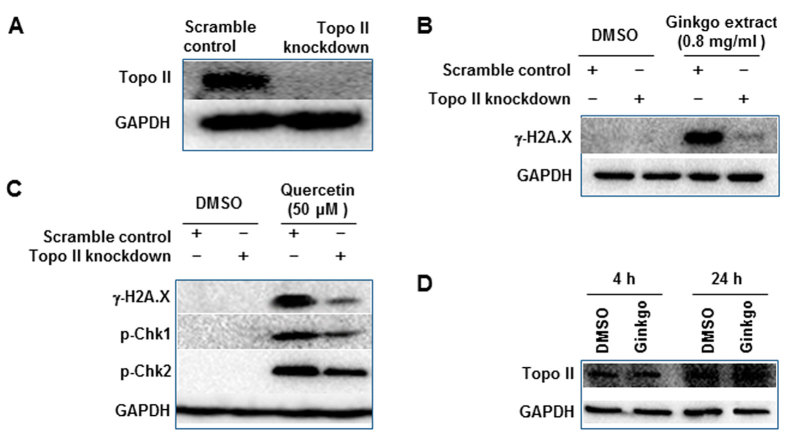
Effect of silencing of Topo II on *Ginkgo biloba* extract- or quercetin-induced DNA damage. (**A**) HepG2 cells stably expressing doxycycline (DOX)-inducible Topo II knockdown and scramble control cell lines were incubated with DOX for 3 days followed by continued culture for another 4 h without DOX; then the Topo II knockdown efficiency was assessed by Western blot. (**B,C**) Topo II knockdown and scramble control cells were incubated with DOX for 3 days and then treated with *Ginkgo biloba* leaf extract at 0.8 mg/ml (**B**) or quercetin at 50 μM (**C**) for another 4 h without DOX. Treated cells were then lysed and subjected to Western blot analyses with antibodies against γ-H2A.X, p-Chk1, and p-Chk2. Similar results were obtained from three repeated experiments. (**D**), HepG2 cells were exposed to *Ginkgo biloba* extract at the concentration of 0.8 mg/ml for 4 h or 24 h. Cells lysis was subjected to Western blot analysis with an antibody against Topo II.

Quercetin’s inhibitory potential on Topo II was demonstrated using an *in silico* approach (Fig. 3) and a cell-free system (Fig. 4A,C), while the inhibitory activity of *Ginkgo biloba* leaf extract was demonstrated in a cell-free system (Fig. 4D). To investigate whether or not Topo II plays a role in *Ginkgo biloba* leaf extract- or quercetin-induced DNA damage in cells, a previously generated doxycycline-inducible Topo II knockdown HepG2 cell line^27^ was used. Topo II was markedly suppressed at the transcriptional level as demonstrated previously^27^, and at a protein level compared to the scramble control as demonstrated by Western blotting in this study (Fig. 8A). There were no difference between Topo II-silenced HepG2 cells and its scramble control cells by the lactate dehydrogenase activity assay, indicating Topo II-silencing did not affect cell viability (Supplementary Fig. 4).

The induction of γ-H2A.X was measured in both scramble control cells and Topo II knockdown cells exposed to *Ginkgo biloba* leaf extract of 0.8 mg/ml or quercetin of 50 μM for 4 h. The Western blotting results showed that silencing of Topo II markedly decreased γ-H2A.X induction caused by *Ginkgo biloba* leaf extract (Fig. 8B) or quercetin (Fig. 8C) compared to the scramble control cells. In agreement with the reduced DNA damage in Topo II knockdown cells, decreased levels of two cell cycle checkpoint proteins (p-Chk1 and p-Chk2) after quercetin treatment were also observed in Topo II-silenced cells compared to the scramble controls (Fig. 8C). A similar study was conducted in Topo I-silencing cells, and no difference was observed between scramble control and Topo I-silencing cells (Supplementary Fig. 5). In addition, the 4-h or 24-h treatments with *Ginkgo biloba* leaf extract did not significantly change the expression of Topo II (Fig. 8D). These data suggest DNA breakage and cell cycle distribution caused by quercetin or *Ginkgo biloba* leaf extract is Topo II-mediated, due to inhibition of Topo II.

## Discussion

Previous genotoxicity studies using the Ames test in *Salmonella typhimurium* and *Escherichia coli*^5^,^28^, micronucleus assays^29^, and the mouse lymphoma gene mutation assay^30^ have demonstrated that *Ginkgo biloba* leaf extract and some of its constituents caused DNA damage. Our previous study also demonstrated that treatment of mouse lymphoma cells with *Ginkgo biloba* leaf extract and its constituents resulted in chromosomal damage; and quercetin and kaempferol, compared to the other six constituents tested, had the greatest impact on the genotoxicity associated with *Ginkgo biloba* leaf extract^6^.

The major components identified in *Ginkgo biloba* leaf extract are flavonoids (ranging from 23–35%) and terpenoids (ranging from 0.17–11.3% for bilobalode, ginkgolide A, ginkgolide B, and ginkgolide C)^1^,^7^,^31^. Of the flavonoids, quercetin, kaempferol, and isorhamnetin are the most abundant. A standardized extract EGb 761, commercially available in European markets, contains 28% flavonoids. The *Ginkgo biloba* leaf extract used in the current study, our previous study^6^, and a part (methods development) of the NTP study^5^ contains 36.9% flavonoids, with the proportion of quercetin, kaempferol, and isohamnetin being 19.6%, 14.5%, and 2.8%, respectively.

Although studies of some flavonoids on topoisomerase inhibition have been performed, whether or not flavonoids (including quercetin) are inhibitors of Topo I or inhibitors of both Topo I and Topo II has been controversial^24^,^25^,^26^,^32^. To our knowledge, a comparison of *Ginkgo biloba* constituents on topoisomerase inhibition has not been conducted. In the present study using the Topo I enzymatic assay in a cell-free system, none of the seven tested constituents of *Ginkgo biloba* extract showed an inhibitory effect on Topo I activity at concentration of up to 1 mM (Fig. 4B). In addition, Topo I does not seem to be involved in DNA damage trigged by quercetin because silencing of Topo I in HepG2 cells did not alter either the induction of γ-H2A.X or p-Chk1 and p-Chk2, DNA damage response cell cycle check point proteins, compared to the scramble control (Supplementary Fig. 5).

Molecular docking studies demonstrated that quercetin has the greatest potential for interacting with Topo II and may thus cause DNA damage in cells (Fig. 3 and Table 1). To confirm the prediction from *in silico* approach, we used a cell-free system with purified human Topo II enzyme and demonstrated that quercetin is the strongest inhibitor (Fig. 4A,C). It should be noted that cell-free systems do not always reflect the biology in the cells. Therefore, in order to determine if Topo II inhibition is a direct cause of DNA damage caused by *Ginkgo biloba* leaf extract or quercetin, Topo II knockdown cells were employed. When Topo II expression in HepG2 cells was silenced, DNA damage was suppressed as shown by the decreased induction of γ-H2A.X (Fig. 8B,C) and the elevated levels of cell cycle checkpoint proteins p-Chk1 and p-Chk1 were also dramatically decreased after quercetin treatment (Fig. 8C). In contrast, silencing of Topo I showed no change on the DNA damaging effect (Supplementary Fig. 5). Collectively, these results serve as direct evidence that inhibition of Topo II, but not of Topo I, plays a critical role in the induction of DNA double strand breaks.

Topoisomerase inhibitors, such as etoposide, inhibit Topo II re-ligation of replicating DNA^17^, but some Topo II inhibitors, such as m-amsacrine, are also DNA intercalators^33^. To determine whether or not quercetin can intercalate into DNA and to rule out that DNA damage is the result of chemical-DNA interaction, a DNA unwinding assay using linearized plasmid pHOT1 DNA was performed to detect changes in the twisting of the duplex helix (Supplementary Fig. 6). While m-amsacrine, a DNA intercalator, decreased the twisting of DNA (increased the supercoiled form DNA), quercetin up to 500 μM did not display DNA intercalating activity (Supplementary Fig. 5). Although one study has reported that quercetin intercalated into DNA^34^, under our experiment conditions, DNA intercalation was not detected. Our results suggest that Topo II inhibition, instead of direct interaction with DNA, is the likely mechanism of quercetin-associated DNA damage.

In the previous study using mouse lymphoma cells, we demonstrated that *Ginkgo biloba* leaf extract and two of its constituents are genotoxic agents, as evidenced by increased mutant frequency, loss of heterozygosity in the mutants, and DNA double-strand breaks^6^. In the present study, the genotoxicity of *Ginkgo biloba* leaf extract was initially confirmed in human lymphoblastoid TK6 cells by using the flow cytometry method of the GreenScreen HC assay (Supplementary Fig. 1). Because liver was reported to be one of the primary target organs of *Ginkgo biloba* leaf extract-induced carcinogenicity in the NTP 2-year carcinogenicity study^5^, in this study human hepatic cells were used to study further the underlying mechanism associated with DNA damage caused by *Ginkgo bilob*a leaf extract and its constituents. Human-derived HepG2 cells have been shown to be useful in evaluating genotoxic agents and elucidating mechanisms^35^,^36^. The capacity for genetic modification, such as overexpressing and silencing genes of interest, is one of the advantages of these cells for in-depth mechanistic studies at the molecular level^37^,^38^,^39^,^40^.

A pharmacological study in heathy volunteers reported that a maximum plasma concentration of 0.431 μM was observed 3 h after 150 mg quercetin was given orally^41^. Various clinical studies on *Ginkgo biloba* products have been conducted^42^,^43^,^44^,^45^; however, pharmacological data are sparse. A rat pharmacokinetic study reported that with a repeated administration of standardized *Ginkgo biloba* extract EGb 761, the plasma concentrations of flavonoids increased 10-fold over that with a single dose administration^46^. Tissue accumulation was also reported in the same study. Additionally, as a dietary supplement, *Ginkgo biloba* leaf extract is likely to be consumed concurrently with other drugs and the plasma concentrations of flavonoids may be increased because of drug-*Ginkgo biloba* interactions^47^,^48^,^49^,^50^,^51^. Considering all the various factors, including inter-individual variation, long-term consumption, tissue accumulation, and drug-*Ginkgo biloba* interaction, the concentrations of 3.13 or 6.25 μM quercetin (about 7- or 14-times the reported plasma concentration of 0.431 μM) exhibiting toxic effect in terms of a stronger inhibitory effect on Topo II activity (Fig. 4C) or DNA damaging effect (Fig. 6) may be clinically relevant.

Despite of controversial conclusions about the beneficial effects of *Ginkgo biloba* products based on numerous clinical trials, *Ginkgo biloba* leaf extracts are consumed in large quantities world-wide. Since DNA damage and Topo II inhibition were observed for *Ginkgo biloba* leaf extract (Figs 1 and 2D), in order to gain information about commercial products, in this study we also evaluated the DNA damaging effects of three *Ginkgo biloba* extract products available in the U.S. market, one of which is similar to the standardized EGb 761 is also available in the European market. One of three commercial products caused DNA damage in human hepatic cells evidenced by the percentage of DNA in tail and level of γ–H2A.X (Fig. 2), whereas the other two did not, indicating a diverse response in term of DNA damage. Because many *Ginkgo biloba* extract products are sold in the United States, it is important to compare these products; thus more commercial products will be purchased for toxicity evaluation and chemical composition analysis and to determine if the flavonoids are the major contributors to DNA damage in these products.

It is worth noting that isorhamnetin, one of constituents contained in *Ginkgo biloba* leaf extract, gave a negative mutagenicity in mouse lymphoma cells in our previous study^6^; however, resulted in a positive response in HepG2 cells in this study (Fig. 5). Following quercetin and kaempferol, isorhamnetin also showed the relatively higher potentials to bind to the Topo II (Table 1). Overall, the results of the present study are consistent with the findings of our previous study that genotoxicity by *Ginkgo biloba* leaf extract may be attributable to the presence of flavonoids, including quercetin and kaempferol^6^. Although it is difficult to determine the precise component(s) that contributes to the toxicity of *Ginkgo biloba* leaf extract in animals because at least 37 components have been identified in a *Ginkgo biloba* leaf extract^5^, and there may be complicated chemical-chemical interactions, monitoring the flavonoid content of *Ginkgo biloba* products might be useful in predicting *Ginkgo biloba*-related DNA damage, considering the mode of action of flavonoids that we have demonstrated and the high flavonoid content of *Ginkgo biloba* leaf extracts.

In summary, we investigated *Ginkgo biloba* leaf extract and seven of its constituents, and observed that the *Ginkgo biloba* leaf extract used in the NTP study, one commercial *Ginkgo biloba* products, and three major flavonoids (quercetin, kaempferol, and isorhamnetin) induce DNA damage in cultured human hepatic cells. We also demonstrated a direct link between Topo II inhibition and DNA damage caused by *Ginkgo biloba* leaf extract and one of the major flavonoids, quercetin. These results indicate that the DNA damage and carcinogenicity of *Ginkgo biloba* leaf extract in animals may be at least in part the result of Topo II inhibition.

## Methods

### Chemicals and reagents

Seven constituents of *Ginkgo biloba* (quercetin, kaempferol, isorhamnetin, ginkgolide A, ginkgolide B, ginkgolide C, and bilobalide) were purchased from Sigma-Aldrich (St. Louis, MO). Fetal bovine serum (FBS) was obtained from Atlanta Biologicals (Lawrenceville, GA). William’s E medium, penicillin, streptomycin, and dimethysulfoxide (DMSO) were purchased from Sigma-Aldrich. Purified human Topo I and II enzymes, Topo I and II assay kits, and unwinding kit were from TopoGen Inc. (Port Orange, FL). *Ginkgo biloba* leaf extract (Lot GBE-50-001003, a tan powdered solid) was a gift from Dr. Po Chan at the National Institute of Environmental Health Sciences (Research Triangle Park, NC). A detailed characterization and analysis of major components are described in the NTP Technical Report 578^5^. Commercial *Ginkgo biloba* extract products were purchased from three American manufacturers who sell herbal extracts.

### Cell culture

The human hepatoma cell line HepG2 was obtained from the American Type Culture Collection (Manassas, VA). The cells were grown in Williams’s E medium supplemented with 10% FBS and antibiotics including 50 U/ml penicillin and 50 μg/ml streptomycin at 37 °C in a humidified atmosphere with 5% CO_2_. The HepG2 cells were adjusted to a concentration of 2 ~ 5 × 10^5^ cells/ml; and 100 μl of the cells were seeded into the wells of 96-well plates, 2 ml into the wells of 6-well plates, 5 ml into 60-mm tissue culture plates, or 10 ml into 100-mm tissue culture plates. All cells were cultured for about 24 h prior to treatment with the indicated concentrations of *Ginkgo biloba* leaf extract, its constituents, or the vehicle control DMSO (final concentration did not exceed 0.1%, vol/vol).

### Cell cycle analysis

After the treatment, 1 × 10^6^ cells were trypsinized and fixed on ice in 1 ml of 70% cold ethanol for 1 h. After washing twice with cold PBS, the cells were resuspended in 0.25 ml of ice-cold PBS containing 0.2 μg/μl RNase A (Qiagen; Valencia, CA) and incubated at 37 °C for 1 h. Propidium iodide (PI; Sigma-Aldrich) was then added to the cells at a final concentration of 10 μg/ml and the cells were further incubated at 4 °C overnight. The analysis of DNA content (cell cycle analysis) was performed the following day on a FACSCanto II flow cytometer (BD Biosciences; San Jose, CA). Data were acquired using FACSDiva software (BD Biosciences) and the cell cycle distribution was determined using ModFit LT 2.0 (Verity Software House Inc; Topsham, ME).

### Cytotoxicity determination

The cytotoxicity of the *Ginkgo biloba* constituents was assessed using lactate dehydrogenase (LDH) assay and MTT assay as described previously^52^,^53^.

### Assay of Topo I-mediated relaxation of supercoiled plasmid

The Topo I enzyme assay was conducted by measuring the relaxation of negatively supercoiled plasmid DNA pBR322 (TopoGen) as described previously^27^.

### Assay of Topo II-mediated kinetoplast DNA (kDNA) decatenation

Topo II enzymatic activity was assayed by measuring decatenation of kDNA (TopoGen) as described previously^27^.

### Comet assay

The alkaline Comet assay, which detects DNA single and double strand breaks, was performed using the method previously described^27^. Briefly, HepG2 cells were seeded into 6-well plates. After overnight of growth, the cells were treated with *Ginkgo biloba* leaf extract (0.2–1.2 mg/ml), commercial *Ginkgo biloba* extract products (0.1%, v/v), or seven constituents of *Ginkgo biloba* (6.25–100 μM) for 4 h. After the treatment, the cells were washed with cold PBS and trypsinized. The assays were performed using a Reagent Kit for Single Cell Gel Electrophoresis Assay according to the manufacturer’s instructions (Trevigen Inc.; Gaithersburg, MD). Three slides were analyzed for all the concentrations and 100 cells per slides were measured. The percentage of DNA in tails was calculated using Comet Assay IV digital analysis system (Perceptive Instruments; Edmunds, UK) and used as the parameter for DNA damage evaluation.

### Western blot analysis

Cells were grown and treated with *Ginkgo biloba* leaf extract, commercial *Ginkgo biloba* extract products, or quercetin in 60-mm tissue culture plates. Western blots were performed using antibodies against γ-H2A.X, p-ChK1 (Ser-345), p-ChK2 (Thr-68), and Topo II (Cell Signaling Technology; Danvers, MA); and GAPDH was used as an internal control (Santa Cruz Biotechnology; Santa Cruz, CA), followed by a secondary antibody conjugated with horseradish peroxidase.

### Molecular docking

The structure of Topo II-alpha was derived from RCSB PDB database (PDB code: 1ZXM). After adding H atoms and removing the co-crystallized ANP, Mg^2+^, and H_2_O, the A chain of Topo II was used for *in silico* docking studies. A similar docking procedure was described previously^54^. In brief, seven constitutes of *Ginkgo biloba* (ligands) were initially charged by the MMFF94 method and then optimized by 1000 steps of steepest-descent energy minimization followed by 1000 steps of conjugate-gradient minimization. Surflex-dock (Syble 8.1, Tripos Inc; St. Louis, MO) was used to dock seven constituents into Topo II. First, three molecular probes including CH4, C=O, and N–H were tessellated in the binding site and optimized based on the Surflex scoring function. Then, the non-redundant probes with high scores were selected to form a so-called “protomol”. The “protomol” obtained was then used to direct the initial placement of the “ligands” during the docking process by a morphological similarity function. Surflex-dock score is an empirical scoring function, which is expressed in -log(Kd) units to represent binding affinity. A higher score represents stronger binding affinity. Consensus score (*C*-score) computed from Surflex-dock score was also calculated to give a consensus evaluation of binding affinities. The *C*-score ranges from 0 to 5 and the larger value represents the better consensus.

### The GreenScreen HC assay

Details of the protocol, data handling, and decision thresholds for the GADD45a-GFP GreenScreen HC genotoxicity assay have been described^22^. Briefly, this assay monitors the expression of the *GADD45a* gene using an in-frame green fluorescent protein (GFP) reporter gene that is hosted by the human lymphoblastoid TK6 cell line. Two strains of TK6 cells, the test strain (GenM-T01) and the non-fluorescent control strain (GenM-C01), were used in 96-well plates. *Ginkgo biloba* leaf extract in a final DMSO concentration of 1% was tested in nine two-fold dilutions (2.44–625 μg/ml) for 46 h. Methyl methanesulfonate (10 and 50 μg/ml) was used as an intra-plate quality control. After the treatments, data were collected on viable cells using a flow cytometer (BD FACSCalibur). A positive response in the flow cytometry method of the assay is defined where the GFP fluorescence is greater than a threshold value of 1.3 times the vehicle-treated control^22^.

### Statistical analysis

Data for Comet assay and cell cycle analysis are presented as the mean ± 1 standard deviation (SD) of at least three independent experiments. Analyses were performed using GraphPad Prism 5 (GraphPad Software; San Diego, CA). Statistical significance was determined by one-way analysis of variance (ANOVA) followed by the Dunnett’s tests for pairwise-comparisons. The difference was considered statistically significant when *p* was less than 0.05.

## Additional Information

**How to cite this article**: Zhang, Z. *et al.* *Ginkgo biloba* leaf extract induces DNA damage by inhibiting topoisomerase II activity in human hepatic cells. *Sci. Rep.* **5**, 14633; doi: 10.1038/srep14633 (2015).

## Supplementary Material

Supplementary Information

## Figures and Tables

**Table 1:** Molecular docking scores.

Compound name	Surflex-score	*C*-score*
Quercetin	7.39	4
Kaempferol	6.69	3
Isorhamnetin	6.18	4
Ginkgolide A	4.82	3
Ginkgolide C	4.38	3
Bilobalide	3.92	2
Ginkgolide B	3.74	2

^*^Consensus score.
