# Supplementary material for: Ginkgo biloba leaf extract induces DNA damage by inhibiting topoisomerase II activity in human hepatic cells
Source: Sci Rep. 2015 Sep 30;5:14633. doi: 10.1038/srep14633 (PMC4588569; doi:10.1038/srep14633)
Supplement: Supplementary Information [file srep14633-s1.pdf]

***Ginkgo biloba* leaf extract induces DNA damage by inhibiting topoisomerase II activity in human hepatic cells**

Zhuhong Zhang[1], Si Chen[2], Hu Mei[3], Jiekun Xuan[2], Xiaoqing Guo[1], Letha Couch[2], Vasily N. Dobrovolsky[1], Lei Guo[2,*], Nan Mei[1,*]

Supplementary Figure 1. **Cytotoxicity (relative cell survival) and genotoxicity (fluorescence induction) of *Ginkgo biloba* leaf extract in human TK6 cells for 46 h in the absence of metabolic activation**. Methyl methanesulfonate (10 and 50 µg/ml) was used as an intra-plate quality control. A positive result is noted where the relative cell survival is less than 90% of the vehicle control for cytotoxicity and the GFP fluorescence is greater than or equal to a threshold value of 1.3 times the vehicle-treated control for genotoxicity.

Supplementary Figure 2. Structures of seven constituents in *Ginkgo biloba* leaf extract.

Supplementary Figure 3. Cytotoxicity of quercetin in HepG2 cells. MTT (A) and lactate dehydrogenase (LDH) activity (B) assays for cell viability were measured after HepG2 cells were treated with the indicated concentrations of quercetin for 4 h or 24 h. Data represent four independent experiments, each performed in triplicate.

Supplementary Figure 4. Effect of down-regulation of Topo II compared with its scramble control cells. HepG2 cells stably expressing doxycycline (DOX) inducible Topo II knockdown and scramble control cell lines were incubated with (DOX) for 72 h followed by continued culture for another 4 h or 24 h without DOX. The lactate dehydrogenase (LDH) activity assay for cell viability was measured (A) and the cell number was counted by using hemocytometer (B) in Topo II-silenced HepG2 cells and scramble control cells. Data represent three independent experiments.

Supplementary Figure 5. **Effect of down-regulation of Topo I on quercetin-induced DNA damage.** HepG2 cells stably expressing doxycycline (DOX) inducible Topo I knockdown and scramble control cell lines were incubated with DOX for 3 days and then treated with quercetin at 50 µM for another 4 h without DOX. Treated cells were then lysed and subjected to Western blot analyses with antibodies against γ-H2A.X, p-Chk1, and p-Chk2. GAPDH was used as a loading control. Similar results were obtained from three repeated experiments.

Supplementary Figure 6. **Effects of quercetin on DNA intercalation.** DNA unwinding was determined by using a DNA unwinding Kit (TopoGen) which is designed to determine if a chemical intercalates to the DNA double-helix leading to DNA unwinding. Briefly, a total of 20 µl reaction mixture containing 10 mM Tris-HCl (pH 7.9), 5% glycerol, 0.1 mM spermidine, 1 mM EDTA, 0.15 M NaCl, 0.1% BSA, 200 ng of pHOT1 DNA, and 2 units of topoisomerase I (TopoGen) was preincutated at 37 °C for 30 min. Quercetin or m-AMSA (positive control) with indicated concentrations was added into the reaction mixture and incubated further for 30 min at 37°C. The reaction was terminated by the addition of SDS (sodium dodecyl sulfate) to 1%, and then was heated at 56 °C for 20 min after 50 µg/ml proteinase K was added. Reaction products were run on a 1% agarose gel in 1xTAE buffer for about 30 min at 100 V/cm and the gel was stained with 0.5 µg/ml ethidium bromide and visualized with a UV light.

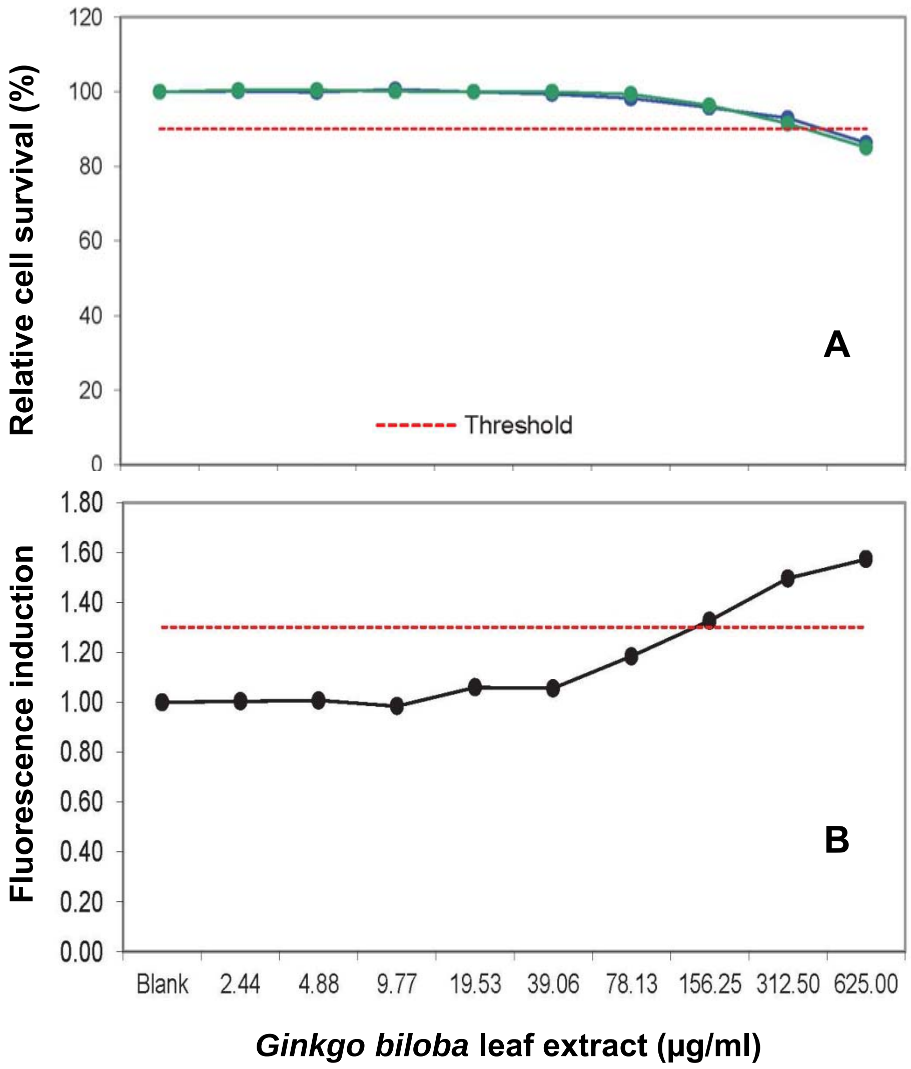

Supplementary Figure 1. **Cytotoxicity (relative cell survival) and genotoxicity (fluorescence induction) of *Ginkgo biloba* leaf extract in human TK6 cells for 46 h in the absence of metabolic activation**. Methyl methanesulfonate (10 and 50 µg/ml) was used as an intra-plate quality control. A positive result is noted where the relative cell survival is less than 90% of the vehicle control for cytotoxicity and the GFP fluorescence is greater than or equal to a threshold value of 1.3 times the vehicle-treated control for genotoxicity.

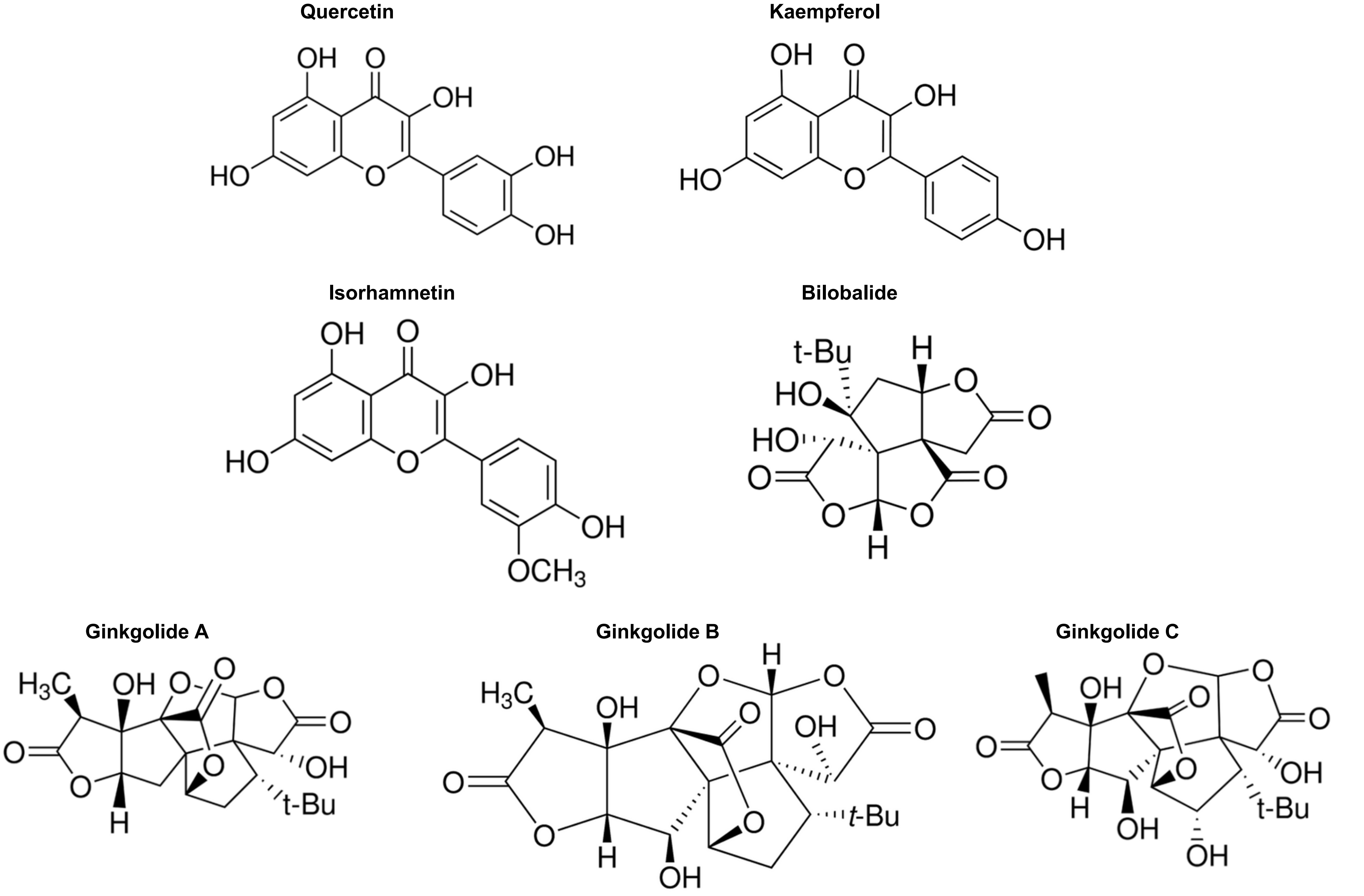

Supplementary Figure 2. Structures of seven constituents in *Ginkgo biloba* leaf extract.

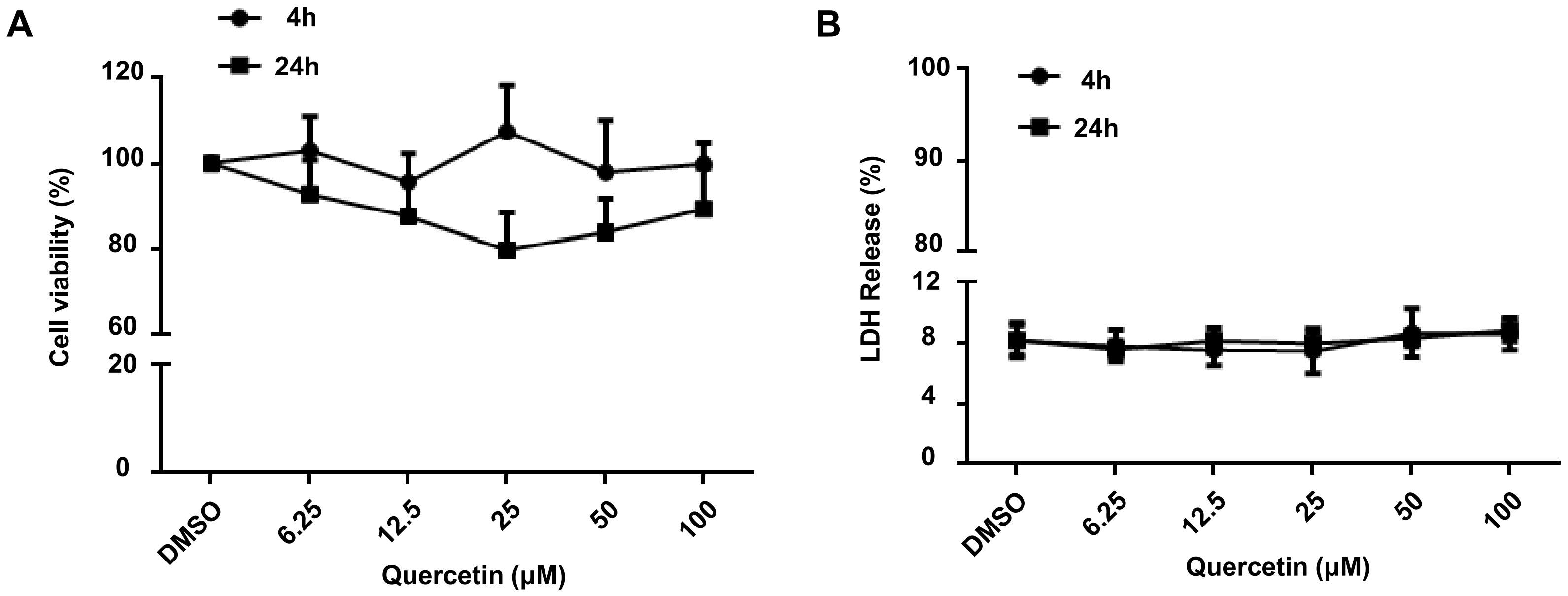

Supplementary Figure 3. Cytotoxicity of quercetin in HepG2 cells. MTT (A) and lactate dehydrogenase (LDH) activity (B) assays for cell viability were measured after HepG2 cells were treated with the indicated concentrations of quercetin for 4 h or 24 h. Data represent four independent experiments, each performed in triplicate.

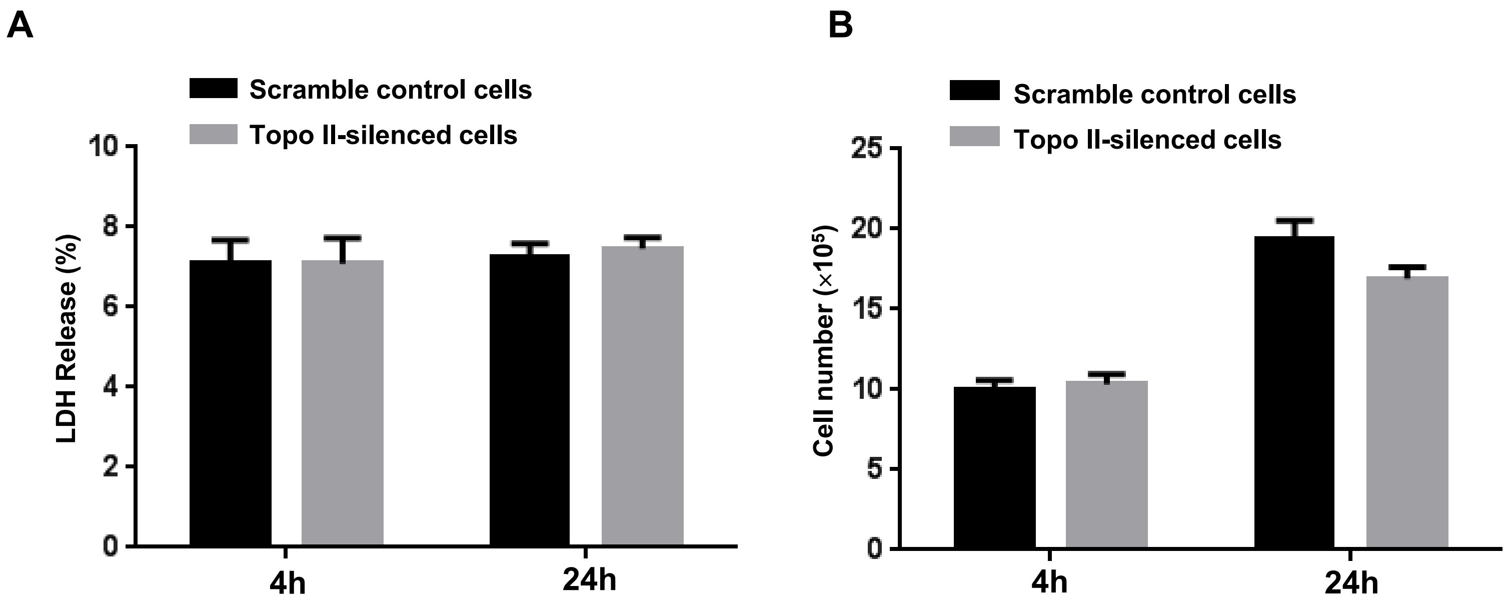

Supplementary Figure 4. Effect of down-regulation of Topo II compared with its scramble control cells. HepG2 cells stably expressing doxycycline (DOX) inducible Topo II knockdown and scramble control cell lines were incubated with (DOX) for 72 h followed by continued culture for another 4 h or 24 h without DOX. The lactate dehydrogenase (LDH) activity assay for cell viability was measured (A) and the cell number was counted by using hemocytometer (B) in Topo II-silenced HepG2 cells and scramble control cells. Data represent three independent experiments.

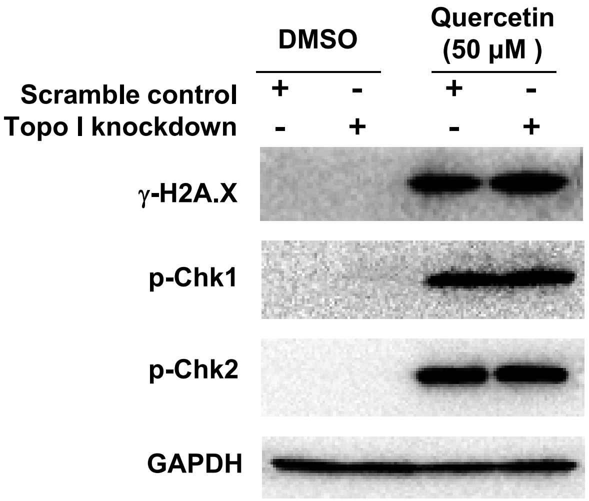

Supplementary Figure 5. **Effect of down-regulation of Topo I on quercetin-induced DNA damage.** HepG2 cells stably expressing doxycycline (DOX) inducible Topo I knockdown and scramble control cell lines were incubated with DOX for 3 days and then treated with quercetin at 50 μM for another 4 h without DOX. Treated cells were then lysed and subjected to Western blot analyses with antibodies against γ-H2A.X, p-Chk1, and p-Chk2. GAPDH was used as a loading control. Similar results were obtained from three repeated experiments.

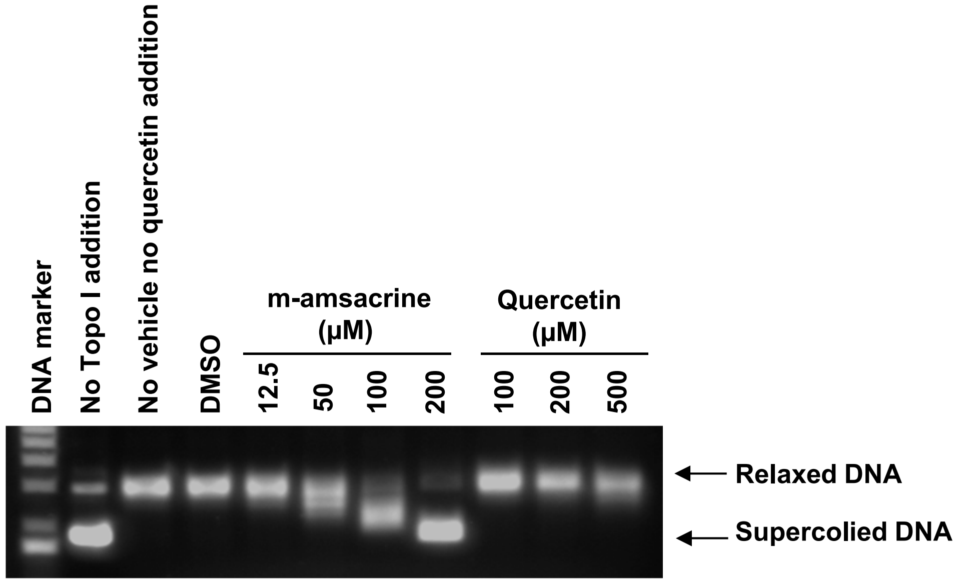

Supplementary Figure 6. **Effects of quercetin on DNA intercalation.** DNA unwinding was determined by using a DNA unwinding Kit (TopoGen) which is designed to determine if a chemical intercalates to the DNA double-helix leading to DNA unwinding. Briefly, a total of 20 μl reaction mixture containing 10 mM Tris-HCl (pH 7.9), 5% glycerol, 0.1 mM spermidine, 1 mM EDTA, 0.15 M NaCl, 0.1% BSA, 200 ng of pHOT1 DNA, and 2 units of topoisomerase I (TopoGen) was preincutated at 37 °C for 30 min. Quercetin or m-AMSA (positive control) with indicated concentrations was added into the reaction mixture and incubated further for 30 min at 37°C. The reaction was terminated by the addition of SDS (sodium dodecyl sulfate) to 1%, and then was heated at 56 °C for 20 min after 50 μg/ml proteinase K was added. Reaction products were run on a 1% agarose gel in 1xTAE buffer for about 30 min at 100 V/cm and the gel was stained with 0.5 μg/ml ethidium bromide and visualized with a UV light.
